# Dilemma of Anticoagulation Therapy in Mild or Asymptomatic COVID-19 Cases

**DOI:** 10.7759/cureus.19291

**Published:** 2021-11-05

**Authors:** Aditya Patel, Folasade Ajayi, Ruhma Ali, Kok Hoe Chan, Jihad Slim

**Affiliations:** 1 Internal Medicine, Saint Michael's Medical Center, Newark, USA; 2 Internal Medicine (Division of Hematology/Oncology), University of Texas Health Science Center at Houston, McGovern Medical School, Houston, USA; 3 Infectious Diseases, Saint Michael's Medical Center, Newark, USA

**Keywords:** sars-cov-2 (severe acute respiratory syndrome coronavirus -2), therapeutic anticoagulation, deep vein thrombosis (dvt), pulmonary embolism (pe), covid-19

## Abstract

Severe acute respiratory distress syndrome coronavirus 2 (SARS-CoV-2) infection can be a life-threatening disease, which has emerged as a public health hazard. Thrombotic events have been reported in hospitalized patients with severe disease however scarce data is available regarding the screening of thromboembolic disease and pulmonary embolism in those with mild or asymptomatic infection. Herein, we describe the development of pulmonary embolism in two asymptomatic patients with COVID-19 and suggest the need for close monitoring and anticoagulation to prevent this life-threatening complication.

## Introduction

The COVID-19 pandemic has resulted in unprecedented increase in mortality globally. COVID-19 is recognized as a multi-organ disease with varied manifestations. Around 4,777,503 deaths have been reported due to this deadly virus as of September 2021 [1]. The clinical spectrum of the disease is variable and ranges from mild symptoms to severe disease with acute respiratory distress syndrome, microvascular thrombosis, cardiac arrest, and death [2]. Venous thromboembolism, in particular, has emerged as an important consideration in the management of patients with COVID-19 [3]. Although the pathogenesis of hypercoagulability in COVID-19 is poorly understood, a prothrombotic state has been reported in patients infected with SARS-CoV-2 with elevation of fibrinogen, prothrombotic particles, and factor VIII [4]. Guidelines for in-hospital treatment of hypercoagulable state in critically ill patients with COVID-19 are available however research is limited for use of anticoagulation in mild COVID-19 patients. Herein, we report two cases of patients with mild COVID-19 who developed pulmonary embolism as a sequela of the disease and raise the question of whether early intervention with anticoagulation should be considered in asymptomatic patients.

## Case presentation

Case 1

**Figure 1:**
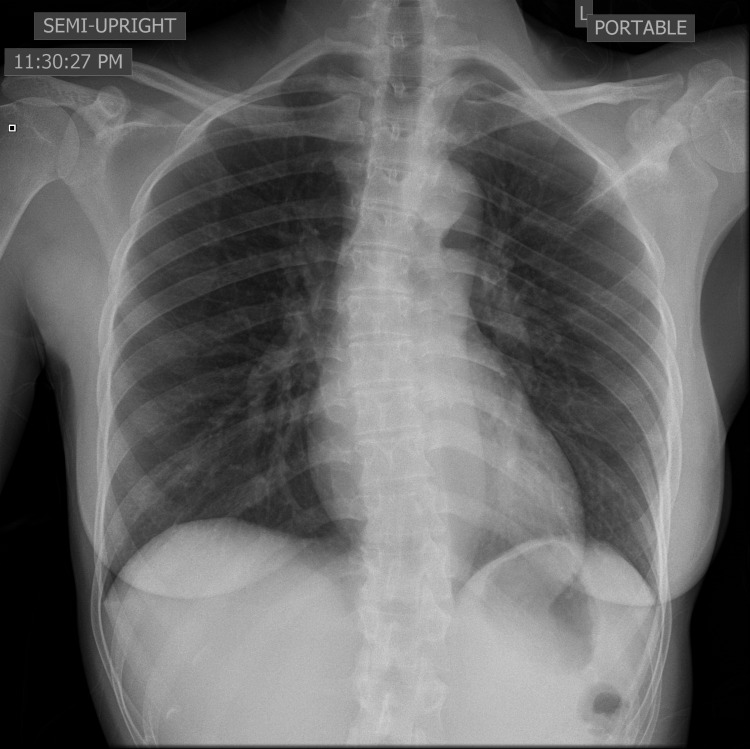
No acute cardiopulmonary changes on the chest X-ray

A 25-year-old female with a past medical history of hypertension and recent SARS-CoV-2 infection presented to the emergency department (ED) complaining of hemoptysis and shortness of breath for two-day duration. She was tested positive for COVID-19 about three weeks earlier after experiencing sore throat. As she was not hypoxic, she was not admitted to the hospital. She reported worsening shortness of breath, constant right-sided chest pain which was worse on inspiration, and occasional palpitations at rest. She denied any leg pain, leg swelling, fever, or dizziness. She reports vaping for the past few years but denies the use of illicit drugs. On admission, vitals were stable, afebrile, and saturating well on room air and on ambulation. Her body mass index was 19.6 kg/m^2^. Electrocardiogram (ECG) showed sinus rhythm with normal axis and no significant ST-T wave changes. Chest X-ray showed no acute cardiopulmonary changes (Figure 1).

Doppler study for bilateral lower extremities showed no evidence of acute deep vein thrombosis (DVT). Initial laboratory showed normal white blood cell and platelets, low hemoglobin, and high D-dimer. Inflammatory markers otherwise were unremarkable. Detailed laboratory parameters are shown in Table 1.

**Table 1:** Detailed laboratory parameters of case 1

Laboratory Parameters	Case 1	Reference Range
White Blood Cell (x10^3^/uL)	5.4	4.4 - 11
Absolute Neutrophil Count (x10^3^/uL)	1.7	1.7 - 7
Absolute Lymphocyte Count (x10^3^/uL)	3.2	0.9 - 2.9
Hemoglobin (g/dL)	10.2	12 - 15.5
Platelets (x10^3^/uL)	337	150 - 450
Creatinine (mg/dL)	1.5	0.6 - 1.2
D-dimer (ng/mL)	1055	0 - 500
Ferritin (ng/mL)	4.8	24 - 336
C-reactive protein (mg/dL)	<0.3	0.0 - 0.8

**Figure 2:**
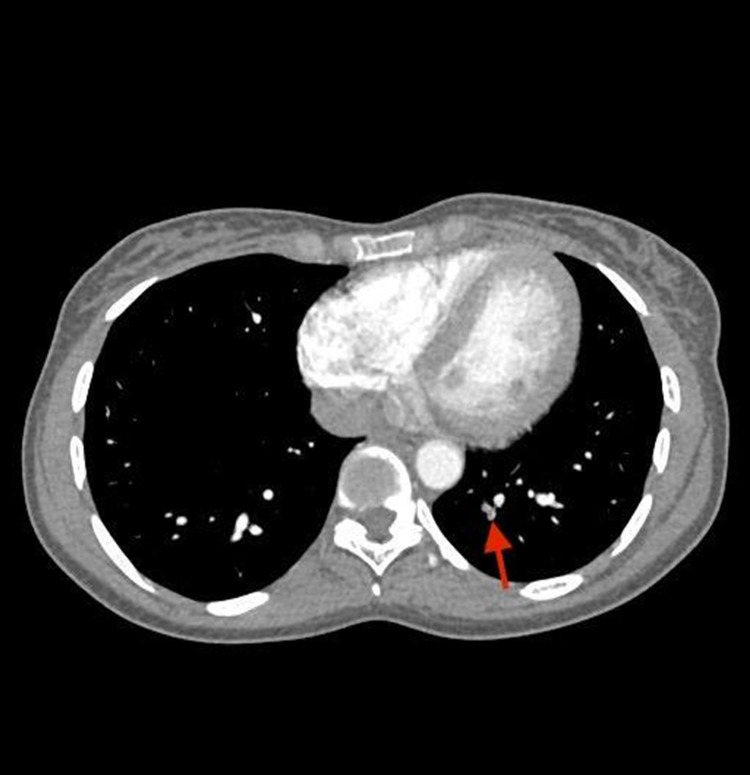
CT angiogram of the chest showed sub-segmental left lower lobe pulmonary embolism

Her nasopharyngeal swab was negative for both SARS-CoV-2 antigen and reverse transcription-polymerase chain reaction. Computed tomography angiography (CTA) showed sub-segmental left lower lobe pulmonary embolism with no evidence of right heart strain as shown in Figure 2.

She was started on heparin drip as per pulmonary embolism protocol. Echocardiography study was normal with no right heart strain. She was clinically stable and was discharged on apixaban 10 mg two times a day for 10 days and then 5 mg two times a day with outpatient follow-up with a hematologist.

Case 2

**Figure 3:**
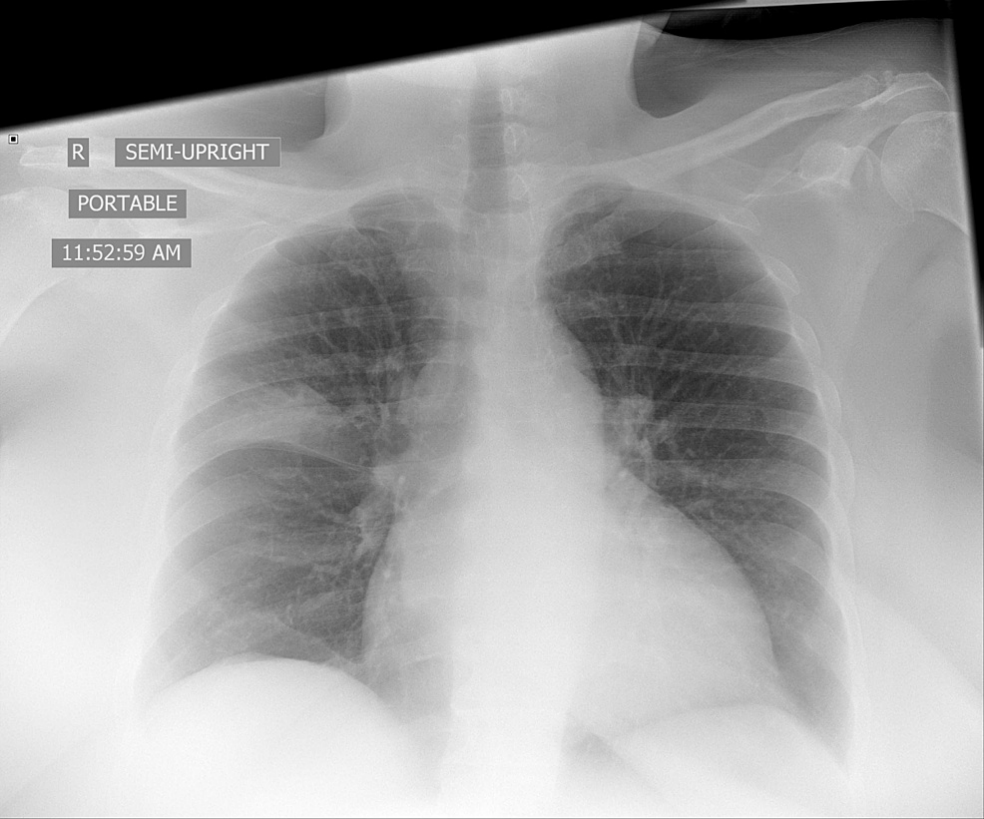
Focal right upper lobar opacity on chest X-ray

A 62-year-old female with a past medical history of hypertension, type II diabetes mellitus, and arthritis presented to the ED with progressive worsening shortness of breath. She was tested positive for SARS-CoV-2 at outpatient but was never hypoxic and was told to quarantine at home without receiving any specific treatment. In the ED, she was afebrile, initial vitals on admission were stable and she was saturating well on room air and on ambulation. ECG revealed sinus rhythm with left ventricle hypertrophy and no ST-T wave changes. Chest X-ray showed focal right upper lobar opacity (Figure 3).

**Figure 4:**
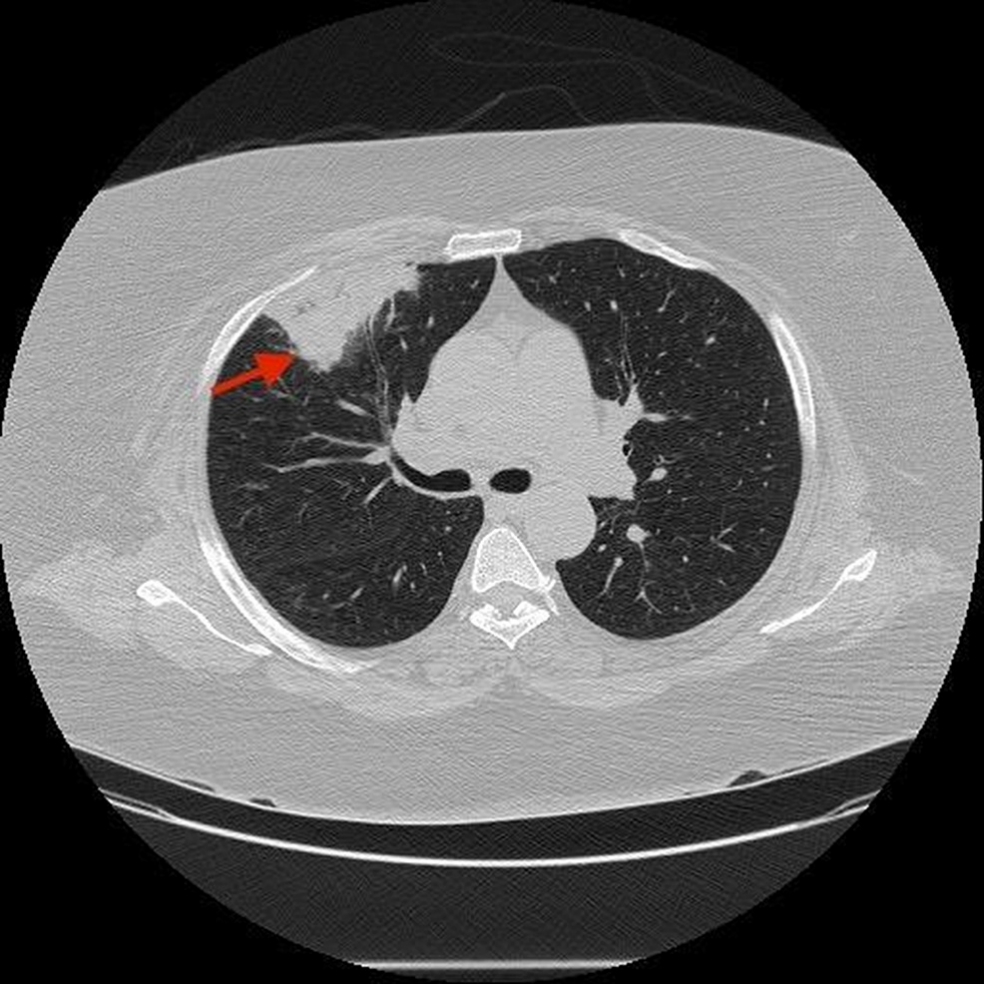
CT chest showed well-demarcated area of consolidation in the peripheral right upper lobe which could represent an area of infarction

Computed tomography (CT) chest without contrast showed a well-demarcated area of consolidation in the peripheral right upper lobe which could represent an area of pneumonia or pulmonary embolism as shown in Figure 4.

Initial laboratory showed normal complete blood counts and very high D-dimer. Inflammatory markers otherwise were unremarkable. Detailed laboratory parameters are shown in Table 2.

**Table 2:** Detailed laboratory parameters of case 2

Laboratory Parameters	Case 2	Reference Range
White Blood Cell (x10^3^/uL)	10.6	4.4 - 11
Absolute Neutrophil Count (x10^3^/uL)	6.5	1.7 - 7
Absolute Lymphocyte Count (x10^3^/uL)	3.1	0.9 - 2.9
Hemoglobin (g/dL)	12.6	12 - 15.5
Platelets (x10^3^/uL)	278	150 - 450
Creatinine (mg/dL)	3.8	0.6 - 1.2
D-dimer (ng/mL)	37,841	0 - 500
Ferritin (ng/mL)	488.6	24 - 336
C-reactive protein (mg/dL)	2.3	0.0 - 0.8

She was tested negative for both SARS-CoV-2 antigen and reverse transcription polymerase chain reaction. Due to the elevated creatinine computed tomography angiography (CTA) was not ordered. The patient was started on anticoagulation based on the CT scan results. However, shortly afterwards the patient was found to be unresponsive with an absent pulse. Cardiopulmonary resuscitation (CPR) was initiated, no shock was given to the patient and she was pronounced dead after failure of return of spontaneous circulation (ROSC).

## Discussion

COVID-19 has emerged as the most consequential global health crisis since the influenza pandemic and has had a catastrophic effect in the world's demographics. Current evidence suggests that SARS-CoV-2 is primarily transmitted through contact and respiratory droplets [5] and the emergence of mutant variants has resulted in increased contagiousness and spread of this virus [6]. COVID-19 infection is associated with a rapid onset of systemic proinflammatory state leading to cytokine storm which is characterized by lymphocytopenia and elevated levels of D-dimer, C-reactive protein, ferritin, lactate dehydrogenase, and interleukins [7]. The clinical sequelae of this disease is characterized by multisystem organ failure, acute respiratory distress syndrome, sepsis, neurological manifestations, thromboembolic disease, and hypercoagulable state [8]. Out of hospital sudden cardiac deaths have also been noted in the patients infected with SARS-CoV-2 virus which can be attributed to rapid decompensation caused by hypoxemic respiratory failure, massive pulmonary embolism, and myocardial infarction. Physical activity limitation increased the incidence of out-of-hospital sudden cardiac death since immobility is a risk factor for thromboembolic disease [9].

The coagulopathy associated with COVID-19 (CAC) is distinct from the sepsis-induced coagulopathy (SIC) and disseminated intravascular coagulopathy (DIC) as it has minimal abnormalities in prothrombin time and platelet count and increase in incidence of venous and arterial thromboembolism [10]. This phenomenon can be due to the fact that COVID-19 causes endothelial injury which predisposes to thrombosis in the arterial and venous system in the absence of atherosclerosis [11]. Some features of CAC overlap with hemophagocytic lymphohistiocytosis (HLH), antiphospholipid syndrome, and thrombotic microangiopathy [10], however, subtle distinctions in clinical and lab findings can be seen like hypofibrinogenemia and hypertriglyceridemia in HLH.

The management of COVID-19-associated coagulopathy has created a unique challenge for health care workers and substantial importance has been given to anticoagulation in hospitalized patients. Guidelines published by the American Society of Hematology (ASH) recommend prophylactic anticoagulation over therapeutic anticoagulation in critically ill patients who do not have suspected or confirmed venous thromboembolism (VTE) [12]. The International Society of Thrombosis and Hemostasis (ISTH) recommends extended post-discharge anticoagulation for patients who meet the high-risk VTE criteria [3]. All guidelines have currently moved away from using therapeutic anticoagulation in hospitalized patients. These guidelines mostly focus on acutely ill hospitalized patients but anticoagulation in asymptomatic or non-hospitalized patients is not well documented. In a recently published article in NEJM, investigators from REMAP-CAP, ACTIV-4a, ATTACC studies showed increased probability of survival with therapeutic anticoagulation over prophylactic anticoagulation in noncritically ill patients [2] with no significant advantage in critically ill patients [13].

In our case series, both patients presented with new-onset shortness of breath after testing positive for COVID-19, 21 days prior to arrival in the ED. Both patients were negative for SARS-CoV-2 RT-PCR in the ED and had mild or largely asymptomatic phases of viral pneumonia and cytokine storm with no need of supplemental oxygen or hospitalization. Both our patients developed thromboembolic disease. These two cases belonged to two different age groups and did not have any other risk factors for thromboembolic events. They were both started on anticoagulation. The prognosis was favorable for the patient in the younger age group while the older patient expired. Our patients represent two ends of the spectrum of VTE after mild COVID-19. The younger patient was discharged on oral anticoagulation with follow-up with a hematologist.

Some of the reported cases of bilateral and extensive PE post mild COVID-19 have shown no need of oxygen supplementation and were treated with oral anticoagulation and close follow-up. Vaccination for COVID-19 and the emergence of new variants have shifted the burden on the healthcare system from critically ill to mild/asymptomatic cases. To date, the focus of anticoagulation has been based on the admitted patient but there is a need for better guidelines and clinical trials for patients with mild/asymptomatic disease to prevent thrombotic complications of this deadly disease. So, follow-up, even after mild COVID-19, may be necessary to address long-term complications. Further research is needed to determine if early out-patient intervention like home-based monitoring or telemedicine is required to identify risk factors like thromboembolic disease in susceptible individuals.

## Conclusions

These two cases describe the development of pulmonary embolism in mild/asymptomatic patients and highlight the need to screen and identify this subset of the population that will benefit from early anticoagulation. Larger future prospective studies and randomized control trials are required to design future guidelines in order to assess the benefits of short-term anticoagulation in patients with COVID-19 with high thrombotic risk and to prevent thrombotic complications in the out-patient setting.
